# Metaplastic breast carcinoma with extensive osseous differentiation

**DOI:** 10.4322/acr.2021.331

**Published:** 2021-09-27

**Authors:** Shalaka Khade, Sudeep Khera, Poonam Elhence, Jeewan Ram Vishnoi

**Affiliations:** 1 All India Institute of Medical Sciences, Department of Pathology and Lab Medicine, Jodhpur, India; 2 All India Institute of Medical Sciences, Department of Surgical Oncology, Jodhpur, India

**Keywords:** Breast diseases, Breast neoplasms, Carcinoma, Ductal, Breast

## Abstract

Metaplastic breast carcinoma is a rare subtype of invasive breast carcinoma. Metaplastic carcinoma with osseous differentiation is exceptionally uncommon. Because of the heterogenous microscopy of the lesion, various clinical and radiological features are observed, leading to diagnostic difficulty. Herein, we present a case of a 43-year-old female with a recurrent breast lump, who was clinically diagnosed as a phyllodes tumor. However, histopathological examination revealed metaplastic carcinoma with extensive osseous differentiation.

## INTRODUCTION

Metaplastic breast carcinomas (MBC) are uncommon invasive carcinomas constituting 0.2% to 1% of all breast cancers.^1^ MBC was identified as a unique entity in the WHO classification of tumors of Breast 2000. The majority of MBCs show triple-negativity for ER, PR, and HER 2neu and are thus associated with poor prognosis.^2^

According to the latest World Health Organization (WHO) system for classifying breast tumors, MBCs are histologically further divided as low-grade adenosquamous carcinoma, fibromatosis-like metaplastic carcinoma, spindle cell carcinoma, squamous cell carcinoma, metaplastic carcinoma with heterologous differentiation and mixed metaplastic carcinoma.^3^ Osseous differentiation in metaplastic carcinoma is very rare.^4^ Herein, we report a case of metaplastic breast carcinoma with osseous differentiation.

## CASE REPORT

A 43-year-old female presented with a firm, non-tender lump in the right breast, which progressed to present size over a period of two months. The mammogram revealed a well-circumscribed, high-density lesion with popcorn-like calcification. Contrast-enhanced computed tomography (CECT) demonstrated a heterogeneously enhancing mass lesion of size 3.2 x 3 x 2.9cm with central necrotic area in upper inner quadrant of the right breast with chunky calcification within the lesion. A clinical diagnosis of Phyllodes tumor was considered.

Biopsy from the lesion revealed a tumor constituting bizarre, pleomorphic cells with atypical nuclei, numerous tumor giant cells, along with osteoid-like material. A diagnosis of malignant neoplasm with osteoblastic differentiation with differential diagnosis of malignant phyllodes with osteoblastic differentiation and metaplastic carcinoma was given.

**Figure 1:**
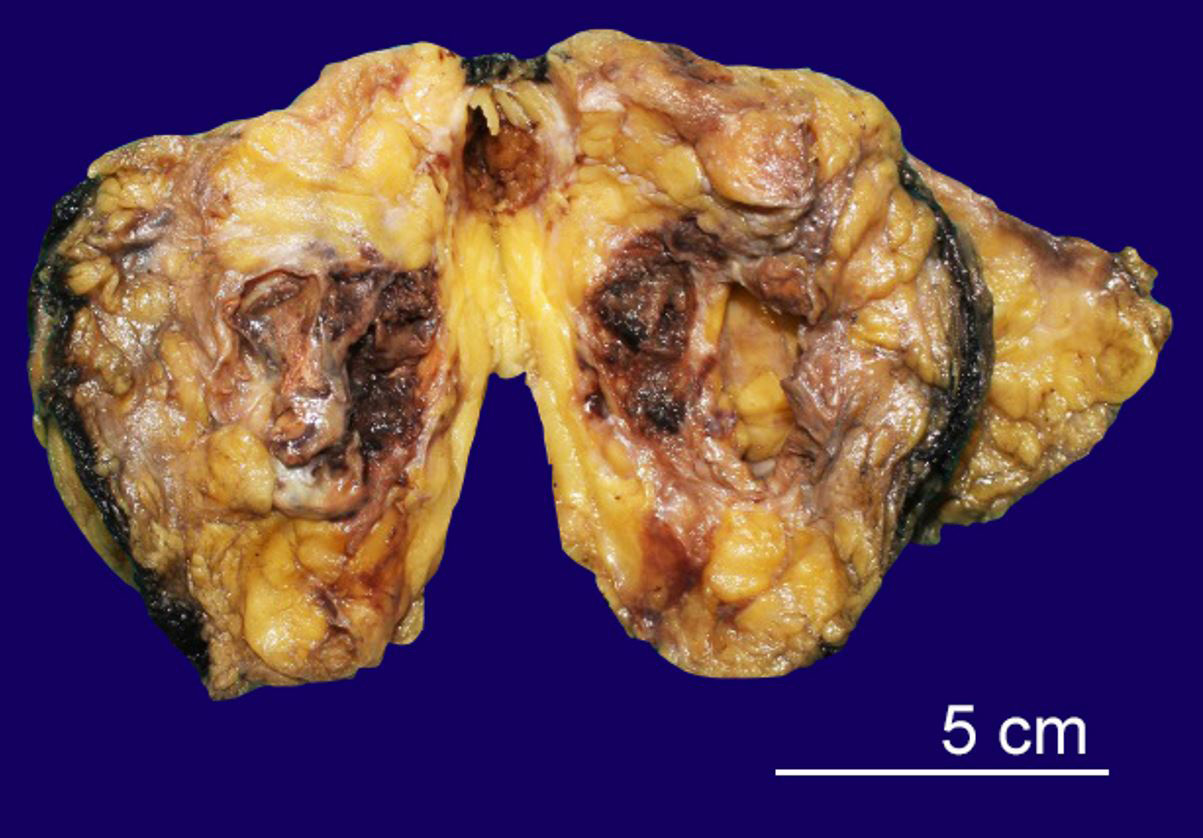
Gross view of the tumor showing multiloculated solid-cystic mass filled with blood.

The patient underwent wide local excision of the lump. The gross appearance of the tumor revealed a multiloculated solid-cystic mass filled with blood (Figure 1).

Frozen section examination revealed a tumor with multiple vascular spaces surrounded by pleomorphic cells and osteoid production evidence. A diagnosis of malignant vascular lesion vs. malignant phyllodes was suggested on cryosection.

**Figure 2:**
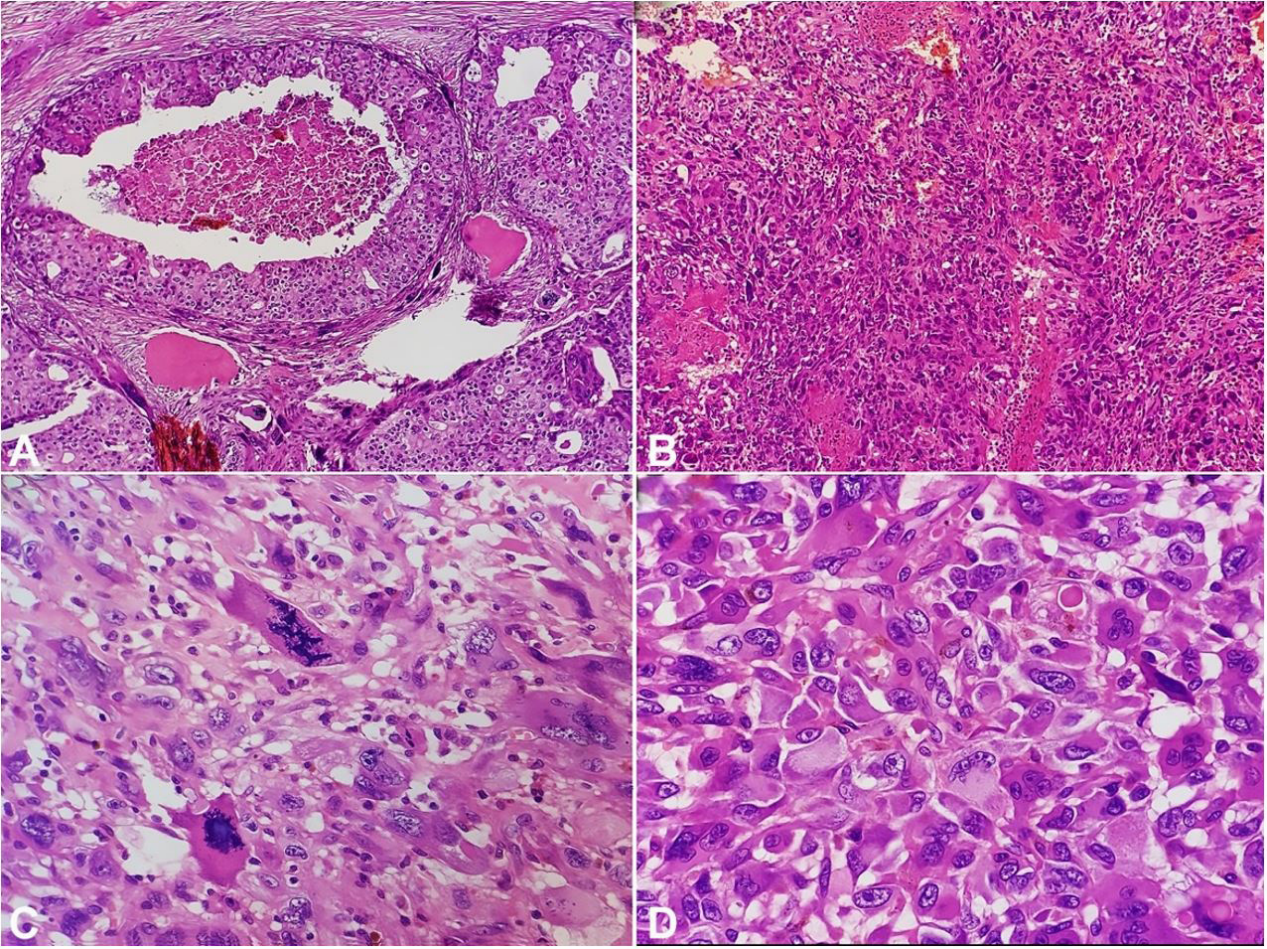
Photomicrograph of the tumor. **A** – Epithelial component, ductal carcinoma in situ with comedonecrosis and cribriform pattern (H&E, ×100); **B** – Malignant stromal component in vague fascicles of spindle cells (H&E, ×100); **C** – Tumor cells with marked nuclear pleomorphism and atypical mitotic figures (H&E, ×400); **D** – Malignant ovoid to spindle shaped cells (H&E, ×400).

**Figure 3:**
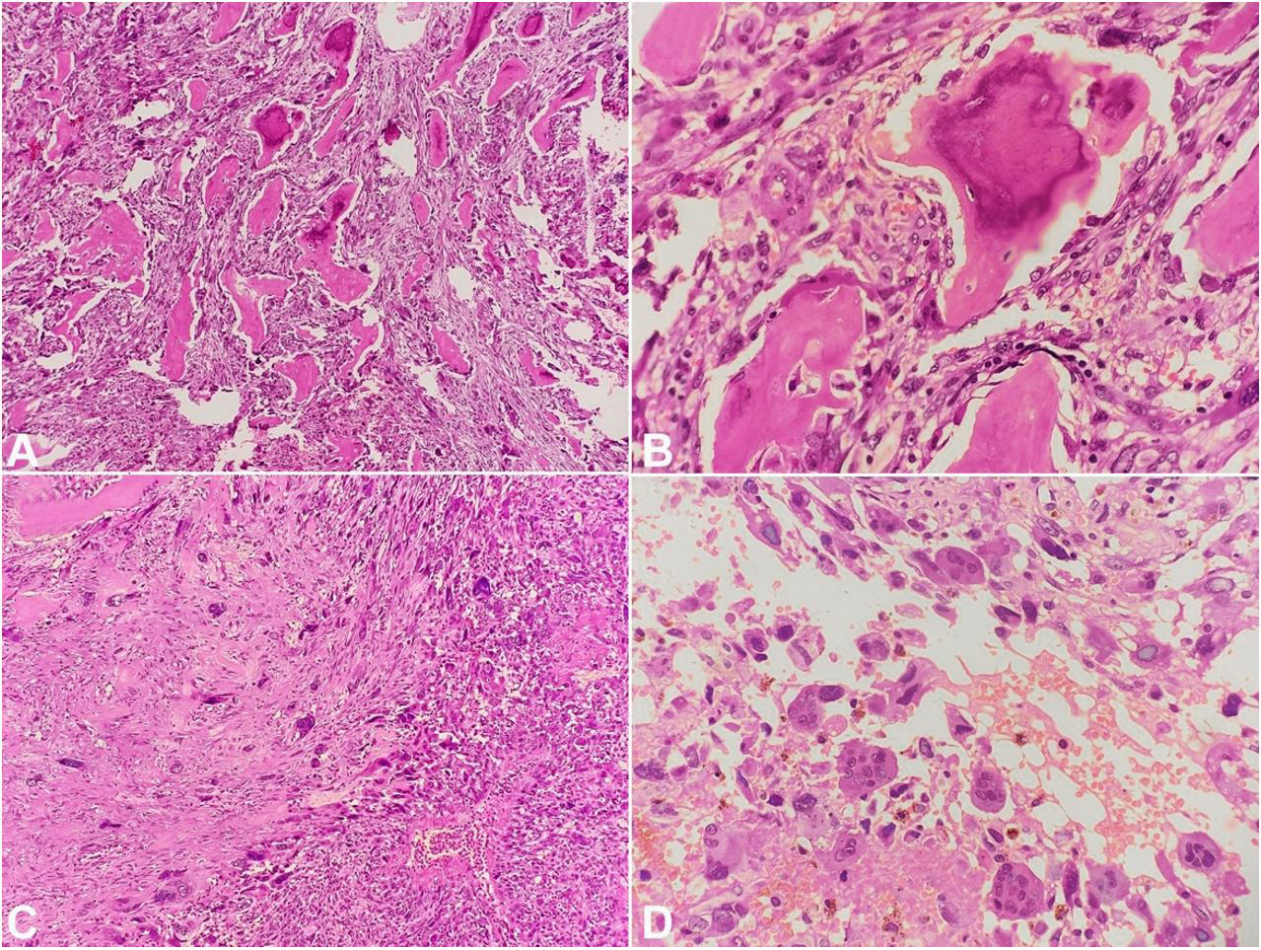
Photomicrograph of the tumor. **A** – Metaplastic breast carcinoma with abundant osteoid formation (H&E, ×100); **B** – High power view showing osseous differentiation (H&E, ×400); **C** – Vague fascicles of spindle cells with numerous tumor giant cells (H&E, ×100); **D** – Numerous osteoclastic giant cells interspersed with malignant cells (H&E, ×400).

Routine microscopic examination revealed an invasive biphasic tumor comprising malignant epithelial and stromal components. The epithelial component was ductal carcinoma-in-situ (DCIS) with high nuclear grade arranged in cribriform and comedonecrosis pattern. Focal apocrine change was noted. The malignant stromal component comprised of vague fascicles of spindle-shaped cells exhibiting marked nuclear pleomorphism, atypia, vesicular nuclei, and 1-2 conspicuous nucleoli. Mitosis of 13/10HPF along with many atypical mitotic figures was noted. Many bizarre, multinucleated and osteoclastic giant cells were noted along with extensive osseous differentiation. Large cystic areas were identified, which were lined by atypical pleomorphic cells filled with hemorrhage. Many bony trabeculae were identified with surrounding osteoblastic rimming (Figure 2 and 3).

**Figure 4:**
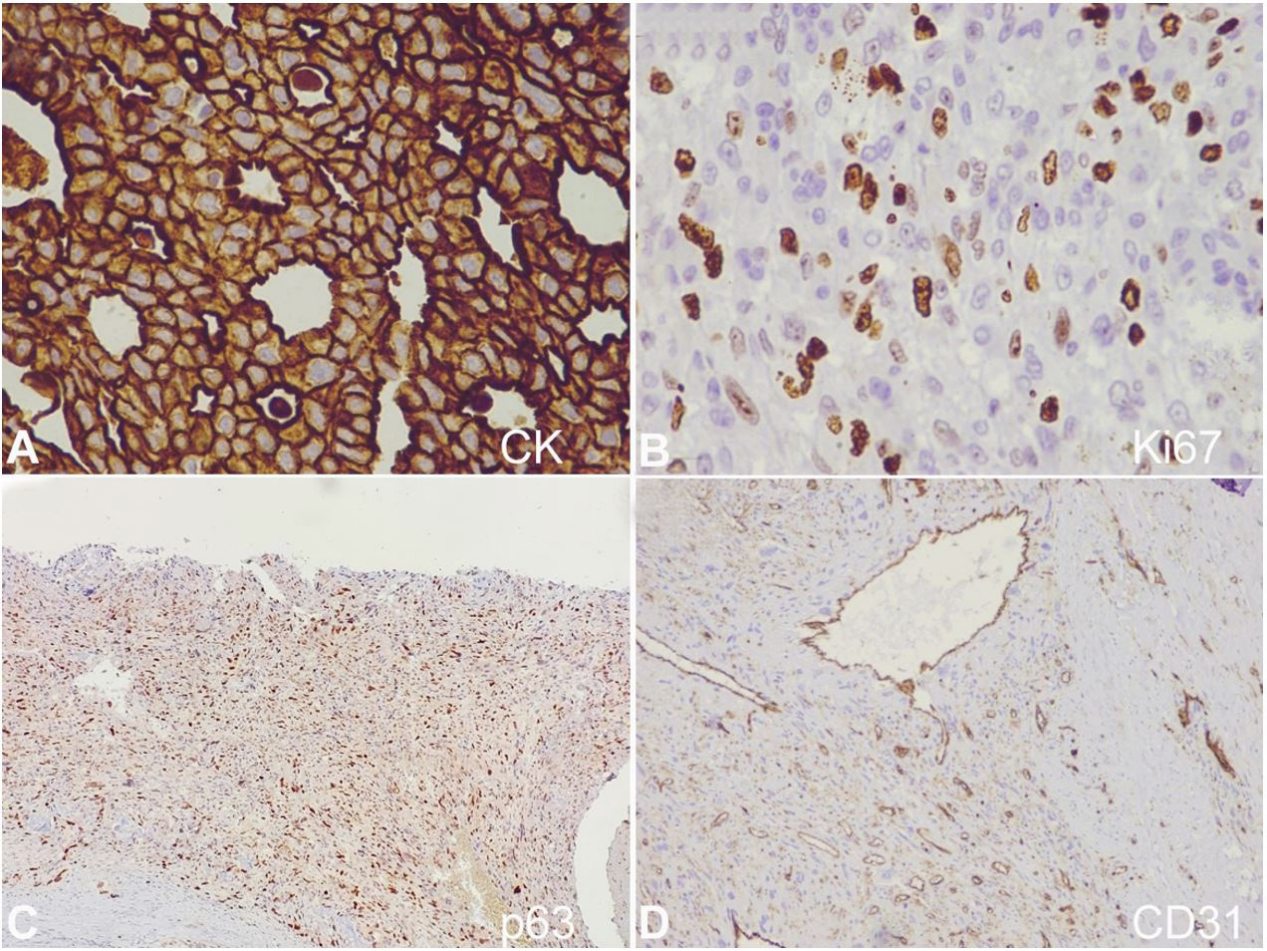
Photomicrograph of the tumor. **A** – CK Epithelial component showing diffuse membranous and cytoplasmic immunoreactivity for cytokeratin (IHC, ×400); **B** – Ki67 proliferation index around 8-10% (IHC, ×400); **C** – p63 positive in mesenchymal component (IHC, ×100); **D** – CD31 highlighting the proliferating vessels between tumor cells (IHC, ×100).

Many proliferating blood vessels were noted interspersed in between the atypical pleomorphic cells highlighted by the CD31 immunohistochemical marker. Immunohistochemical staining demonstrated a strong diffuse membranous and cytoplasmic positivity for Cytokeratin (CK) in the DCIS component. p63 was positive in the mesenchymal component and focally positive in the DCIS component. Ki-67 proliferation index was around 8-10%, and the tumor cells were triple-negative for ER, PR, and Her2neu. CD31 was negative in the tumor cells (Figure 4).

The postoperative period of the patient was uneventful. The patient received four cycles of Adriamycin and Cyclophosphamide followed by four cycles of taxane-based adjuvant chemotherapy. No recurrence occurred during the 6-month follow-up period.

## DISCUSSION

MBCs are a rare subgroup of ductal carcinoma with varied heterogeneity and were first described in 1973.^5^ Median age at which MBCs present ranges from 48 to 59 years.^2^ These are usually greater than 2cm and rapidly growing masses.^6^ MBCs are known to exhibit macroscopic calcification on mammogram and color doppler can manifest a blood-rich lesion; both characteristics were present in the present case.^1^^,^ 4

Microscopically, the mesenchymal components include chondroid, osseous, rhabdomyoid, and sometimes neuroglial elements. The osseous component is a scarce form, accounting for only 0.2% of all breast carcinomas.^1^^,^ 4 Inadequate sampling can lead to misdiagnosis on core needle biopsy and fine-needle aspiration cytology.^7^ The sarcomatous component of MBCs can resemble other spindle cell neoplasms like malignant phyllodes, fibrosarcoma, myoepithelial carcinoma, malignant fibrous histiocytoma, etc. Adequate sampling also helps find the DCIS component which, is strongly supportive of metaplastic carcinoma, thus, avoiding misinterpretation.^7^ Definitive diagnosis of MBC can appropriately be made on excisional biopsy specimens. 1

Immunohistochemical confirmation of tumor epithelial component is necessary for the diagnosis of metaplastic carcinoma. CK5/6, CK14, CK(AE1/AE3), and 34βE12 are commonly used markers. Most of the MBCs demonstrate immunopositivity for p63 and CK, which distinguishes them from fibromatosis.^1^

Metaplastic carcinoma with mesenchymal differentiation shows various patterns and combinations, the admixture of cartilaginous and osseous differentiation being most common. Atypia ranging from bland to overtly malignant features can be noted. Infrequently, osteoclast-type tumor giant cells along with areas of stromal hemorrhage can be seen.^8^ Presence of metaplastic elements, including chondroid, osteoid, and intervening spindle cell component, are associated with poor prognosis in MBCs, whereas predominant carcinomatous component, benign heterologous component, and absent intervening stroma are good prognostic factors.^2^

MBCs are less commonly associated with lymph node involvement and have earlier distant metastasis, thus associated with poorer prognosis.^9^ Some studies in the literature have stated that MBCs tend to have a hematogenous spread instead of lymphatic spread and thus resulting in lung and bone metastasis.^3^

Surgery remains the main modality of treatment for MBC. Adjuvant radiotherapy can help in decreasing the rate of recurrence as well as the mortality rate. However, some studies have found MBCs to be chemoresistant.^2^

## CONCLUSION

MBC with osseous differentiation is a rare subtype of metaplastic carcinomas of the breast, which presents as a circumscribed mass lesion usually with calcifications. The clinical characteristics are varied, making the diagnosis difficult. Being a triple negative carcinoma and with frequent hematogenous spread to lung and bone, it is associated with poor prognosis. Furthermore, it is known to have increased chances of recurrence and resistance to chemotherapy.

## References

[1] Zhou X, Wu X, Wang L (2021). Metaplastic breast carcinoma: a retrospective study of 26 cases. Int J Clin Exp Pathol.

[2] Yahaya J, Mremi A (2020). Metaplastic carcinoma of breast: a report of two cases. Oxf Med Case Reports.

[3] Jia Y, He C, Liu L (2019). A retrospective study of the imaging and pathological features of metaplastic breast carcinoma and review of the literature. Med Sci Monit.

[4] Salih AM, Kakamad FH, Saeed YA, Muhialdeen AS (2017). Metaplastic breast carcinoma with osseous differentiationA rare case report. Int J Surg Case Rep.

[5] Surenkok S, Tahberer E, Cinkaya A, Kodaz H, Deger A (2018). Metaplastic breast cancer: a case report. J Pak Med Assoc.

[6] Luo K, Wang X (2020). Bone in the breast: a case report of a metaplastic breast cancer with osseous differentiation. Radiol Case Rep.

[7] Hasbay B, Bolat FA, Aytaç HÖ, Aslan H, Purbager A (2020). Metaplastic carcinoma of the breast: analysis of 38 cases from a single institute. Turk Patoloji Derg.

[8] McMullen ER, Zoumberos NA, Kleer CG (2019). Metaplastic breast carcinoma update on histopathology and molecular alterations. Arch Pathol Lab Med.

[9] Taghipour Zahir S, Javannejad M (2017). Metaplastic breast carcinoma with chondroid differentiation: a rare variant of infiltrative carcinoma in a 38-year-old woman. BMJ Case Rep.

